# Selective Intestinal Decontamination as a Method for Preventing Infectious Complications (Review)

**DOI:** 10.17691/stm2020.12.6.10

**Published:** 2020-12-28

**Authors:** A.L. Barsuk, E.S. Nekaeva, L.V. Lovtsova, A.L. Urakov

**Affiliations:** Associate Professor, Department of General and Clinical Pharmacology; Privolzhsky Research Medical University, 10/1 Minin and Pozharsky Square, Nizhny Novgorod, 603005, Russia;; Head of Admission and Consultation Department, Clinical Pharmacologist, University Clinic; Privolzhsky Research Medical University, 10/1 Minin and Pozharsky Square, Nizhny Novgorod, 603005, Russia;; Associate Professor, Head of the Department of General and Clinical Pharmacology; Privolzhsky Research Medical University, 10/1 Minin and Pozharsky Square, Nizhny Novgorod, 603005, Russia;; Professor, Head of the Department of General and Clinical Pharmacology; Izhevsk State Medical Academy, 281 Kommunarov St., Izhevsk, 426034, Udmurt Republic, Russia; Leading Researcher, Department of Modeling and Synthesis of Technological Processes Udmurt Federal Research Center, Ural Branch of the Russian Academy of Sciences, 34 Tatyany Baramzinoy St., Izhevsk, 426067, Udmurt Republic, Russia

**Keywords:** selective intestinal decontamination, infectious complications of burn injuries, burn injury

## Abstract

Infectious complications are the most common cause of death in patients with severe burns. To date, there is no generally accepted method for preventing such complications in burn injury. One of the possible prevention options is selective intestinal decontamination (SID). This method is based on the enteral administration of non-absorbable antimicrobial agents. The preventive effect of SID involves inhibition of intestinal microflora translocation through the mucous membranes, inasmuch as studies demonstrate that endogenous opportunistic microorganisms are a common cause of infectious complications in various critical conditions.

The SID method was originally developed in the Netherlands for patients suffering from mechanical injury. Antimicrobial drugs were selected based on their high activity in relation to the main endogenous opportunistic pathogens and minimal activity against normal intestinal microflora components. The combination of polymyxin (B or E), tobramycin, and amphotericin B with intravenous cefotaxime was chosen as the first SID regimen. Other regimens were proposed afterwards, and the application field of the method was expanded. In particular, it became the method of choice for prevention of infectious complications in patients with severe burn injury.

Clinical studies demonstrate efficacy of some SID regimens for preventing infectious complications in patients with thermal injury. Concomitant administration of SID and systemic preventive antibiotics and addition of oropharyngeal decontamination increases the method efficacy. SID is generally well-tolerated, but some studies show an increased risk of diarrhea with this preventive option. In addition, SID increases the risk of developing antibiotic resistance like any other antibiotic regimens.

## Introduction

Despite the constant decrease in both the incidence and mortality from thermal injury, treatment of burns remains an urgent medical and social problem. Relatively high mortality rates, long hospitalization, and high levels of disability in the injured are characteristic of thermal injury [1–5]. On average, about 180,000 people in the world die each year from thermal injury and its consequences [6]. The main factors of mortality are the large size of the lesion and elderly age of the injured. In the first 48 h after injury, deaths are most often caused by burn shock and inhalation injury. In the later periods, the main causes of death are infectious complications: sepsis, pneumonia, etc. In general, sepsis is the most common cause of death in burn injury [1, 7–9].

Antimicrobial drugs are normally applied to prevent infectious complications. However, studies of effective antibiotic prophylaxis in burns show conflicting results. Therefore, international as well as national recommendations for treatment of thermal injury do not include this method [10–12]. Despite this, there is continuous research investigating various regimens for the prevention of infectious complications with antimicrobial drugs and the most promising options for such prevention are selective intestinal decontamination (SID) and oropharyngeal decontamination. The rationale for their administration is proven translocation of endogenous microflora organisms of these locations under various critical conditions, including burn injury. Studies demonstrate that endogenous microflora particularly often becomes the etiological factor of microbial complications. The pathogenesis of bacterial translocation involves decreased gastrointestinal motility, increased permeability of the mucous membranes, increased rates of bacterial growth in the intestine, decreased immune responsiveness, and toxemia. Thermal inhalation injury, prolonged intubation, and mechanical ventilation are also important factors for the development of endogenous infection [13–18].

## Development of selective intestinal decontamination method

Initially, this method was developed mainly for patients not with thermal, but mechanical injury. In the early 1980s, Chris Stoutenbeek, a resuscitator at the University Hospital of Groningen (the Netherlands), took an interest in the problem of infectious complications in patients with severe mechanical trauma. He organized a group of resuscitators, microbiologists, and other specialists who began to investigate the cultures of microorganisms isolated from such patients. The endogenous flora of the intestine and pharynx appeared to play a leading role among the pathogens. By that time, oncologists of the Groningen University Hospital had already developed several regimens for antibiotic prophylaxis of infectious complications in patients with leukemia. It involves application of non-absorbable antibiotics, primarily neomycin, amphotericin B, polymyxin in combination with trimethoprim + sulfamethoxazole [19, 20].

A group of researchers led by Stoutenbeek conducted a pilot non-controlled study in which patients with severe mechanical injuries (32 persons), patients after cardiac surgery (5 persons), patients with sepsis (18 persons), staying in the intensive care unit (ICU) for more than 5 days, received a combination of polymyxin E, trimethoprim + sulfamethoxazole and amphotericin B. In addition, the patients’ oral cavities were treated with chlorhexidine solution. However, the microbiological examination of the oropharyngeal and intestinal flora prior to prophylaxis revealed wide prevalence of strains, namely representatives of the *Pseudomonas* and *Acinetobacter* genera sensitive to polymyxins, but resistant to the combination of trimethoprim + sulfamethoxazole. As a result, this regimen failed to demonstrate high prophylactic efficacy: almost a quarter of patients (22%) developed pneumonia. Besides, patients poorly tolerated the regimen: several people developed thrombocytopenia or allergic reactions. It is known that the combination of trimethoprim + sulfamethoxazole may cause dangerous systemic reactions, and the risk of these reactions is increased in severely ill patients due to decreased renal function and unpredictable intestinal absorption of medication components. Trimethoprim in a therapeutic dose leads to a decrease in potassium excretion, which may provoke hyperkalemia [21–23].

Stoutenbeek et al. developed a different decontamination regime. They administered polymyxin as a non-absorbable antibiotic highly active against most intestinal bacteria and *Pseudomonas aeruginosa*. It should be noted that polymyxins do not affect the anaerobic flora — the main component of normal intestinal microflora. Polymyxin E was recommended at a dose of 400 mg/day, polymyxin B — 300 mg/day. Moreover, the authors decided to combine polymyxin with aminoglycoside due to insufficient activity of polymyxin in relation to representatives of the *Proteus*, *Morganella*, and *Serratia* genera. Drugs highly active against *Pseudomonas aeruginosa* were selected from the group of aminoglycosides, because polymyxins, the second component of the regimen, are inactivated by fecal enzymes and lose their antipseudomonal activity in feces, which leads to insufficient effect on *Pseudomonas aeruginosa* in feces. The combination of polymyxin + aminoglycoside was selected not only due to the synergistic effect on *Pseudomonas aeruginosa* but also because of practically absent cross-resistance in relation to these groups of antibiotics. The choice was made from three antipseudomonal aminoglycosides, namely gentamicin, tobramycin, and amikacin, taking into account that the antipseudomonal activity of these drugs is quite similar, though tobramycin, which was finally opted for, is the most stable in feces and at a dose not exceeding 500 mg/day has almost no effect on normal intestinal microflora. Results of other clinical and experimental studies [22, 24–28] demonstrate the ability of polymyxin/tobramycin combination to reduce the levels of bacterial endotoxins in feces. The authors of the study administered tobramycin at a dose of 320 mg/day.

The use of antibacterial agents leads to increased growth of fungi of the *Candida* genus, which necessitated administration of an antifungal medication. There were proposed polyene antimycotics in high doses: amphotericin B at a dose of 2 g/day (4 doses) or nystatin at a dose of 848 IU/day (8 doses). These high doses of polyenes are determined by their significant inactivation by fecal enzymes and are justified by low absorption in the gastrointestinal tract [22, 29–31].

To prevent staphylococcal infection, the authors of the technique proposed parenteral cefotaxime as a drug with high antistaphylococcal activity and a well-proven minimal effect on the normal microflora of the gastrointestinal tract. Besides, cefotaxime creates high concentrations in saliva and bile. It was decided not to administer oral or parenteral vancomycin as Methicillinresistant Staphylococcus aureus (MRSA) was quite rare in the late 70s — early 80s of the XX century [22, 26, 32–34].

As a result, the regimen used in the study was as follows: polymyxin E (100 mg) + amphotericin B (500 mg) + tobramycin (80 mg) in the form of a 10 ml suspension through a nasogastric tube 4 times a day. Cefotaxime was administered in standard doses (50–100 mg/kg/day) intravenously during the first 4 days of prophylaxis [15, 22, 26].

The results of the first study of this SID regimen efficacy (in this article — standard SID regimen) were published in 1983 [35]. In this study, SID was performed in 63 patients (the main group), while the control group consisted of 59 people without medicamentous prophylaxis. All patients were in the ICU for at least 5 days. In the control group, 48 patients (81%) developed an infection, and there were 94 episodes. The most common infections were those of the lower respiratory tract (35 cases). The mortality rate in this group was 5 people (8%). In the main group, infection was detected only in 6 patients (8%). All cases were associated with the development of pneumonia, and their analysis revealed exogenous microorganisms as the main pathogens. All patients of the main group survived.

Later, this group of authors conducted a multicenter randomized study of this method, involving 401 patients with severe mechanical injuries (at least 16 scores on Hospital Trauma Index-Injury Severity Score) [36]. According to the described method, 201 patients received SID, while the control group consisted of 200 patients. Although the study revealed no significant effect of SID on the overall mortality (20.9% in the SID group and 22% in the control group), this method demonstrated the ability to significantly reduce the level of respiratory tract infections in this category of patients. The upper respiratory tract infections were observed in 30.9% of cases in the SID group and in 50% of patients in the control group, pneumonia — in 9.5 and 23% of cases, respectively, tracheobronchitis — in 25.9 and 40%, respectively. The overall level of infectious complications decreased significantly — 48.8 and 61.1%, respectively. However, in this study, SID failed to reduce the incidence of urinary tract infections, blood system infections, and wound infections.

To date, there have been published the results of many randomized clinical trials (RCTs) of SID efficacy. They show the ability of this method to significantly reduce the incidence of infectious complications in ICU patients [37–43], in surgical interventions on the gastrointestinal tract [44–49] and the cardiovascular system [15, 50], in liver transplantation [51–53] and allogeneic hematopoietic stem cell transplantation [54], during cytostatic therapy [55, 56]. In 2003, SID method developers published an article [57] where they argued, referring to the studies available at that time, that SID efficacy in prevention of infectious complications exceeded in many clinical situations the efficacy of systemic antibiotic prophylaxis and strategies based only on hygiene measures (barrier and isolation technologies). The authors underlined that a greater success of such prevention should be achieved by using drugs not only orally or through a nasogastric tube, but also applying them to treat the oropharyngeal zone and rectum. Besides, the authors referred to studies demonstrating SID cost-effectiveness.

## The use of selective intestinal decontamination in burn patients

A meta-analysis of 21 RCTs of SID efficacy in treatment of various pathologies in critically ill patients (4902 patients in total) [58] showed that SID significantly reduced overall mortality while having a minor effect on the mortality associated with infectious complications. According to the meta-analysis, 18 patients should have received SID to prevent one death. Notably, this meta-analysis was the first to include data from one RCT of SID efficacy in patients with severe burns [59]. SID was performed according to the standard method proposed by Stoutenbeek et al. In addition to SID, patients received oropharyngeal decontamination with a paste containing polymyxin E + amphotericin B + tobramycin. This double-blind study involved 107 patients (53 in the SID group and 54 in the placebo group) over 14 years old with a burn surface area of more than 20%. In the ICU, mortality was significantly lower in the SID group than in the placebo group and amounted to 9.5% and 27.8%, respectively; the risk of developing ventilator-associated pneumonia was also significantly lower. To prevent one death, 5 patients had to receive SID.

Later, the authors of this study demonstrated the ability of SID to reduce the degree of respiratory and hematological dysfunction in this category of patients, which also seems to play a significant role in increasing the survival rates in burn patients [60].

Standard SID regimen was used in a retrospective study of this prevention method efficacy in patients with severe (more than 30% of body area) burns, conducted in the Burn Center of Beverwijk (Netherlands) [61]. The SID group included 31 patients. Treatment results of 33 patients hospitalized before applying SID method were used as the control. SID significantly reduced the incidence of burn wound colonization by *Pseudomonas spp*. (29% in the SID group vs 61% in the control group) and *Enterobacteriaceae spp.* (10% in the SID group vs 73%, respectively). A similar decrease in colonization by gram-negative organisms was found in urine and gastric aspirates. There were fewer respiratory infections in the SID group (6.5 vs 27.3% in the control group), and only 1 patient developed septicemia, while in the control group there were 8 such patients (3.2 vs 24.2%). Mortality in the SID group was also lower — 1 patient compared to 7 in the group without prophylaxis. After 2 years, the authors published the results of another study comparing the efficacy of standard SID regimen (34 patients) and SID with the addition of intranasal mupirocin (33 patients). The new regimen demonstrated higher efficacy in relation to colonization of wound fluid, sputum, and gastric aspirate by *Staphylococcus aureus* [62].

When performing SID, it is necessary to take into account the regional characteristics of significant pathogen prevalence. For example, MRSA strains have been rarely encountered in the Netherlands so far [34], and classical SID regimen developers consider this method unsuitable for geographic areas with high MRSA prevalence [36]. Moreover, MRSA is an important pathogen causing infectious complications in burn patients in many countries [63–66] and, in particular, in Russia [67–69]. A large prospective study conducted in Spain [70] involved two groups: 402 patients for whom only barrier and isolation measures were used to prevent contamination of burns from the environment (group 1), and 375 patients (group 2) receiving SID according to standard regimen + enteral vancomycin (500 g orally 4 times a day, 4% vancomycin paste for intranasal and oropharyngeal application). In group 1, mortality rate was 18.2%, in group 2 — 10.9% (the differences were statistically significant). Notably, the lesion area averaged 30.3% in group 1, while it was 25.61% in patients of group 2, which could also affect the different mortality rates in the groups. The overall rate of MRSA isolation was significantly reduced in the enteral vancomycin group. For example, 115 isolates (28.6%) of this microorganism were identified in group 1, and 25 (6.7%) in group 2. There was decreased isolation of the microorganism determined in the study in all locations: wound fluid, blood, tracheal aspirate. The use of vancomycin did not lead to increased frequency of isolation of Vancomycin-resistant enterococcus (VRE) strains and decreased sensitivity of MRSA strains to glycopeptides.

A number of studies focused on the efficacy of SID regimens different from those proposed by Stoutenbeek et al. (polymyxin + tobramycin + amphotericin B + cefotaxime). For example, in work [71] there was no intravenous administration of antimicrobial drugs. However, their results as well as the results of some other studies and systematic reviews demonstrate that administration of non-absorbable antibiotics alone is less effective in preventing infectious complications than mixed SID regimens [72, 73].

An early study [74] focused on the effect of prophylaxis with non-absorbable oral antibiotics (neomycin + erythromycin + nystatin without parenteral administration of antimicrobial drugs) on the microflora of burn wounds. It was found that microbial colonization occurred after an average of 4 days without antibiotic prophylaxis, and with antibiotic prophylaxis — after 19 days. Besides, the study revealed a tendency towards lower occurrence of bacterial colonization of tissues in the burn wound area (the level of bacterial contamination was 10^5^ microbial bodies). The number of such patients was 2 times lower among those who received antibiotic prophylaxis. However, a placebo-controlled randomized trial [75] did not confirm the efficacy of neomycin + erythromycin + nystatin prophylaxis regimen in 30 patients with burns over 20% of the body. In this RCT, the mean time before burn wound colonization was 6.1 days in the antibiotic prophylaxis group and 6.7 days in the placebo group. Moreover, earlier colonization of *Pseudomonas aeruginosa* was noted in the wound fluids of patients receiving antibiotics. In blood cultures of patients treated with antibiotics, representatives of the *Enterobacteriaceae* family were relatively less common, except for *Proteus spp.*, which, on the contrary, were detected more often during antibiotic prophylaxis. Besides, enterococci were more often detected in the blood of such patients.

In another clinical and microbiological study [76], 91 patients with severe burns (over 25% of the body surface area) received oral polymyxin B as antibiotic prophylaxis. In 63 patients, co-trimoxazole and amphotericin B were administered in addition to polymyxin. The addition of co-trimoxazole significantly reduced colonization of the burn wound by enterobacteria — from 71 to 11%, by Proteus — from 36 to 0%, amphotericin B reduced the frequency of yeast colonization from 39 to 10%.

In a controlled study [77], 256 patients with extensive burns (lesion areas of more than 15% in children and more than 25% in adults) were randomized into three groups. Patients of group 1 received no antibiotic prophylaxis (control), patients of group 2 received SID (polymyxin + co-trimoxazole + nystatin), in group 3, allopurinol (a xanthine oxidase inhibitor) was administered in addition to SID, which was in line with the data of an experimental study, demonstrating the ability of allopurinol to prevent translocation of microorganisms from the gastrointestinal tract in shock. SID significantly reduced the risk of wound contamination with intestinal bacteria. SID remained effective over a period of 4 weeks. Besides, prophylaxis significantly reduced mortality and length of hospital stay in surviving patients. Allopurinol had no influence on these effects of SID.

Another double-blind RCT [78] studied the efficacy of the polymyxin E + tobramycin + amphotericin B regimen without the use of systemic antimicrobial drugs for prevention of infectious complications in children under 15 years of age with severe burns (the average lesion area was 67% in the main group and 58% in the placebo group). In this study, the use of non-absorbable antibiotics did not lead to a significant effect on microbial colonization of burn wounds, sputum, nasogastric aspirates, and feces. This prevention strategy did not significantly affect the incidence of pneumonia, sepsis, and other infectious complications as well as the concentration of inflammatory markers such as interleukins (IL-1β, IL-6, IL-10, and TNF-α). Notably, the frequency of diarrhea in the prophylaxis group was significantly higher than in the placebo group, which allowed the authors to conclude that prophylaxis with non-absorbable medications was ineffective and poorly tolerated in children with severe burns. However, this study is remarkable for a small number of participants: 11 patients in the main group and 12 in the placebo group.

In RCT [79], 30 patients with severe burns (burn lesion area was 30–50%) were divided into two equal groups. Patients of the main group received antibiotic prophylaxis with non-absorbable antimicrobial drugs (amikacin + miconazole + polymyxin M-sulfate) and intravenous ciprofloxacin during the 1^st^ week of treatment, while the control group received no antibiotic prophylaxis. Prevention significantly reduced the level of bacteremia (4 cases in group 1, and 12 cases in the control), the overall frequency of infectious complications (2 cases in group 1, 8 in the control), and also reduced bacterial colonization of wounds (9 and 14, respectively). In the main group, mortality was 26.7%, while being 2 times higher in the control. The length of hospital stay in surviving patients was significantly shorter in the antibiotic prophylaxis group. The effect of prophylaxis on IL-6 levels was insignificant.

## The safety of using selective intestinal decontamination

The safety of SID has not been fully studied yet [31]. As a rule, prescribed drugs are well tolerated. Polymyxins, polyenes, aminoglycosides administered orally have low bioavailability and do not lead to undesirable systemic effects. However, concentration of some drugs administered orally as part of SID can be quite high in critically ill patients. Monitoring of tobramycin concentration in blood serum of such patients showed that it could reach toxic values (>2.0 mg/L) in some cases, which is likely to be associated with increased permeability of the intestinal barrier and decreased liver and kidney function [80–82]. There are few reports of antibiotic-associated diarrhea due to SID. There is evidence of an increased risk of complications associated with *Clostridium difficile*, including pseudomembranous colitis, when using SID regimens without vancomycin or metronidazole [78, 83].

One of the important issues related to SID is its effect on the levels of antibiotic resistance of microorganisms. Studies of this aspect of using SID technology show conflicting results [37, 40]. For example, a 16-year study revealed no increase in the frequency of detecting multidrug-resistant forms of microorganisms due to long-term use of SID in ICU of some Spanish hospitals [84]. Some studies show paradoxical results: SID is associated with lower rates of colonization by resistant gram-negative bacteria and no effect on MRSA and VRE [37, 85]. Meta-analysis [86] revealed an increase in the number of resistant strains of gram-negative bacilli to polymyxin and third-generation cephalosporins due to SID application. On the other hand, SID did not affect the increase in the levels of gram-negative bacilli resistance to aminoglycosides and fluoroquinolones, as well as the frequency of detecting MRSA and enterococci strains resistant to vancomycin. Another meta-analysis of applying the SID method during 20 years revealed increased resistance of microorganisms to cephalosporins — by 7.9%, to polymyxins — by 3.5%, and to ciprofloxacin — by 8% [87].

Investigation of the effect of SID on the intestinal microbiota demonstrated an increase in the level of resistance genes to aminoglycosides of commensal microorganisms. The authors of the study [88] note that in this case, these genes might be transferred to opportunistic and pathogenic bacteria. A high level of resistance genes to aminoglycosides of commensal microorganisms persists for some time after cessation of SID [89]. A large study carried out in the Netherlands (11,997 participants) [90] demonstrated that the use of SID (6116 persons) or oropharyngeal decontamination (5881) in ICU patients led to a significant increase in aminoglycoside-resistant strains of gram-negative microorganisms in feces: by the average of 7% during 1 month in patients receiving SID, and by 4% in those who received oropharyngeal decontamination. Adding enteral vancomycin to SID increases the risk of VRE appearing in the intestinal microflora [91]. However, there are data confirming the absence of bacterial resistance growth during SID in burn patients [70, 71]. Nevertheless, practice of using any antimicrobial agents leaves no hope — the widespread use of SID and oropharyngeal decontamination will definitely increase antibiotic resistance and change the structure of pathogenic microorganisms [92, 93].

## The future of selective intestinal decontamination in Russia

In our country, studies investigating preventive efficacy of SID are rare and the use of this method seems to be very limited in Russia. For example, there were studies on SID efficacy in prevention of infectious complications in acute destructive pancreatitis [94–96], in correction of intestinal dysbiosis in patients with lung cancer combined with chronic obstructive pulmonary disease [97], and in prevention of sepsis in patients with hepatic insufficiency [98], in patients with progressive odontogenic phlegmon [99]. There is evidence of applying SID regimens with fusidic acid before various surgical interventions [100]. In the domestic literature, there has been found no evidence of applying SID and/or oropharyngeal decontamination in burn injury. It should be noted that the use of SID method is limited in Russia for certain reasons including external ones: several basic drugs traditionally used for this type of antibiotic prophylaxis are unavailable in Russia in the enteral form. For example, according to the State Register of Medicines, only polymyxin B is registered among injectable polymyxins, tobramycin and other aminoglycosides are not registered as forms for enteral administration, amphotericin B is available only in forms for parenteral administration and as a dermatological ointment. Those scarce domestic studies investigating SID efficacy involved enteral administration of amikacin, fluconazole, and ciprofloxacin [94, 95], clindamycin, metronidazole, and kanamycin together with the Normospectrum probiotics [97], fluconazole, and gentamicin [98]. In these works, special attention is paid to the enteral use of injectable aminoglycosides (the so-called off-label administration). Apart from injectable polymyxin B, vancomycin is available for enteral administration in our country [101].

## Conclusion

Clinical studies have shown that SID method is effective in preventing infectious complications in various critical conditions, including burns. This option of antibiotic prophylaxis can increase survival rates of patients and reduce the length of hospital stay. However, the evidence for SID efficacy in burn injury is far from exhaustive. The obvious disadvantage of the method is the lack of clear practice guidelines for using SID in burns. Studies demonstrate that SID does not lead to rapid development of microorganism resistance in burn patients, though the widespread use of this method will predictably contribute to antimicrobial drug resistance. When using this method, it is necessary to take into account the specificity of pathogens of infectious complications in a particular pathology for a particular geographic area. For example, to prevent infectious complications in burn injury in regions with high MRSA prevalence (such as Russia), it is recommended to include enteral vancomycin in SID regimen. Oropharyngeal decontamination increases SID efficacy. The use of non-absorbable antimicrobial drugs alone appears to be less effective than their concomitant administration with parenteral antibiotics.

In Russia, SID is extremely rarely used to prevent infectious complications in various pathologies, including burns. One of the important reasons for this is the lack of enteral aminoglycosides, polymyxins, and amphotericin B on the domestic pharmaceutical market. However, polymyxin B and aminoglycoside drugs for parenteral use are available in our country. In our opinion, off-label administration of these drugs for SID is possible in thermal injury, because there are evidence-based data from clinical studies, systematic reviews, and meta-analyses, confirming preventive efficacy of this method in reducing the risk of development and severity of infectious complications in this pathology. Moreover, enteral administration of these drugs is relatively safe as they are practically not absorbed in the gastrointestinal tract. The results of studies carried out in our country and demonstrating SID efficacy may serve as the basis for introduction of this administration route in the guidelines for parenteral aminoglycosides and polymyxin B or the basis for registration of special enteral forms of these drugs.
